# Multiplex PCR−Based Next-Generation Sequencing and Global Diversity of Seoul Virus in Humans and Rats

**DOI:** 10.3201/eid2402.171216

**Published:** 2018-02

**Authors:** Won-Keun Kim, Jin Sun No, Seung-Ho Lee, Dong Hyun Song, Daesang Lee, Jeong-Ah Kim, Se Hun Gu, Sunhye Park, Seong Tae Jeong, Heung-Chul Kim, Terry A. Klein, Michael R. Wiley, Gustavo Palacios, Jin-Won Song

**Affiliations:** Korea University, Seoul, South Korea (W.-K. Kim, J.S. No, S.-H. Lee, J.-A. Kim, J.-W. Song);; Agency for Defense Development, Daejeon, South Korea (D.H. Song, D. Lee, S.H. Gu, S. Park, S.T. Jeong);; 65th Medical Brigade/Medical Department Activity−Korea, Seoul (H.-C. Kim, T.A. Klein);; US Army Medical Research Institute of Infectious Disease, Fort Detrick, Maryland, USA (M.R. Wiley, G. Palacios)

**Keywords:** next-generation sequencing, multiplex PCR, orthohantavirus, Seoul virus, hantavirus, viruses, phylogenetic analysis, global diversity, humans, rats, reverse transcription PCR, RT-PCR, real-time quantitative PCR, South Korea

## Abstract

Seoul virus (SEOV) poses a worldwide public health threat. This virus, which is harbored by *Rattus norvegicus* and *R. rattus* rats, is the causative agent of hemorrhagic fever with renal syndrome (HFRS) in humans, which has been reported in Asia, Europe, the Americas, and Africa. Defining SEOV genome sequences plays a critical role in development of preventive and therapeutic strategies against the unique worldwide hantavirus. We applied multiplex PCR–based next-generation sequencing to obtain SEOV genome sequences from clinical and reservoir host specimens. Epidemiologic surveillance of *R. norvegicus* rats in South Korea during 2000–2016 demonstrated that the serologic prevalence of enzootic SEOV infections was not significant on the basis of sex, weight (age), and season. Viral loads of SEOV in rats showed wide dissemination in tissues and dynamic circulation among populations. Phylogenetic analyses showed the global diversity of SEOV and possible genomic configuration of genetic exchanges.

Hantaviruses (order *Bunyavirales*, family *Hantaviridae*, genus *Orthohantavirus*) pose a worldwide public health threat and are the causative agents of hemorrhagic fever with renal syndrome (HFRS) in Eurasia and hantavirus pulmonary syndrome in the Americas (*1*). HFRS is caused mainly by Old World hantaviruses, such as Hantaan virus (HTNV), Seoul virus (SEOV), Dobrava–Belgrade virus, and Puumala virus, that are transmitted to humans by inhalation of dust contaminated with rodent excreta (saliva, urine, and feces) or bite by an infected rodent. Annually, 150,000 cases of HFRS are reported (case-fatality rate range <1%–15%) (*2*). Clinical signs and symptoms include headache, myalgia, abdominal and back pain, nausea, vomiting, diarrhea, proteinuria, thrombocytopenia, hemorrhage, and renal failure (*3*). The typical disease course consists of 5 phases: febrile, hypotensive, oliguric, diuretic, and convalescent: the phases vary in length from several hours to several days. A difficulty in diagnosis is the extensive incubation period from the time of exposure to the onset of symptoms, which might be as long as 50 days. There are no effective vaccines or antiviral agents against hantavirus infection.

SEOV has a negative-sense, single-stranded, tripartite RNA genome (*4*). The large (L) segment encodes an RNA-dependent RNA polymerase, the medium (M) segment encodes 2 membrane glycoproteins (Gn and Gc), and the small (S) segment encodes a nucleoprotein. Brown rats (*Rattus norvegicus*) and black rats (*R. rattus*) are the primary reservoir hosts of SEOV and have a worldwide distribution (*5*,*6*).

SEOV infections have been reported in Asia, Europe, the Americas, and Africa (*7*–*12*). HFRS caused by SEOV is responsible for 25% of clinical cases and is a mild form with a case-fatality rate of <1% in Asia (*13*). Recently, an outbreak of SEOV-induced HFRS was reported in the United Kingdom among rat owners, breeders, and distributors of the pet animal market (*14*). In the United States, outbreaks of SEOV infections occurred in 11 states in 2017; there were 17 confirmed SEOV-infected patients (*15*,*16*). SEOV was identified in New York, New York, and is considered an urban public health threat (*17*).

Whole-genome sequencing of SEOV is a prerequisite for tracking SEOV infections and evaluating disease risks for development and implementation of preventive and therapeutic strategies. Acquisition of viral genome sequences plays a critical role in surveillance, identification, and risk mitigation of outbreaks of virus infection (*18*). Next-generation sequencing (NGS) is a potent tool for defining virus genome sequences. However, an obstacle for obtaining virus genomic information is ultra-low virus RNA loads in the clinical specimens. To enrich the low amount of viral RNA, we developed a multiplex PCR–based NGS that showed high coverage of HTNV genome sequences from HFRS patients (*19*).

In this study, we collected 1,269 *R. norvegicus* rats in an urban HFRS-endemic area in South Korea during 2000–2016. We report a robust strategy for whole-genome sequencing of SEOV and provide useful insights into epidemiologic characteristics and phylogeographic diversity of a unique worldwide hantavirus.

## Materials and Methods

### Ethics

Human samples were provided after informed consent was obtained. The study was approved and conducted in accordance with ethicals guidelines for the Korea University Institutional Animal Care and Use Committee. Live trapping of rats at US military training sites and installations was approved by US Forces Korea in accordance with regulation 40–1 (Prevention, Surveillance, and Treatment of Hemorrhagic Fever with Renal Syndrome). Rats were humanely killed by cardiac puncture, and tissues were collected under isoflurane anesthesia in accordance with procedures approved by Korea University Institutional Animal Care and Use Committee protocol #2010–212.

### Sample Collection

We tested retrospective HFRS patient serum samples obtained from the Korea Bank for Pathogenic Viruses (Seoul, South Korea). We collected *R. norvegicus* rats during 2000–2016 by using collapsible live-capture traps (Tomahawk Live Trap Co., Hazelhurst, WI, USA, and H.B. Sherman, Tallahassee, FL, USA). Traps were set at intervals of 1–2 m and examined early the next morning over a 1–2-day period at US Army training sites. For the US Army Garrison in Seoul, we used baited live capture traps (Tomahawk Live Trap Co.) or glue boards. Captured rats were submitted to the 5th Medical Detachment/Medical Command Activity–Korea, US Army Garrison (Yongsan, Seoul), and then transported to the College of Medicine, Korea University (Seoul), where they were held in a Biosafety Level 3 laboratory until processing. Live rats were humanely killed by cardiac puncture under isoflurane anesthesia and identified to species by using morphologic criteria and PCR, when required. Serum, lung, spleen, kidney, and liver tissues were collected aseptically and frozen at −70°C until used.

### Indirect Immunofluorescence Antibody Test

We used an indirect immunofluorescence antibody (IFA) test for serum samples from HFRS patients and live rats. We initially diluted samples 1:32 in phosphate-buffered saline and then tested them for IgG against SEOV. We applied diluted serum samples to slides containing SEOV-infected Vero E6 cells fixed with acetone and incubated wells at 37°C for 30 min. The slides were washed, fluorescein isothiocyanate–conjugated goat antibody to human and rat IgG (ICN Pharmaceuticals, Laval, Quebec, Canada) was added, and slides were incubated at 37°C for 30 min. We then washed the slides again and examined them for virus-specific fluorescence by using a fluorescent microscope (Axio Scope; Zeiss, Berlin, Germany).

### Real-Time Quantitative PCR

We performed real-time quantitative PCR (qPCR) for total RNA by using the high-capacity RNA-to-cDNA Kit (Applied Biosystems, Carlsbad, CA, USA) in a 10-μL reaction mixture containing 1 µg of total RNA. We used an SYBR Green PCR Master Mix (Applied Biosystems) in a StepOne Real-Time PCR System (Applied Biosystems). We performed reactions at 95°C for 10 min, followed by 45 cycles at 95°C for 15 s, and then 1 cycle at 60°C for 1 min. Primer sequences specific for SEOV S segments were SEOV-S719F: 5′-TGGCACTAGCAAAAGACTGG-3′ and SEOV-S814R: 5′-CAGATAAACTCCCAGCAATAGGA-3′.

### Reverse Transcription and Rapid Amplification of cDNA Ends PCR

We extracted total RNA from serum or lung tissues of seropositive samples by using TRI Reagent Solution (Ambion Inc., Austin, TX, USA). We synthesized cDNA by using the High Capacity RNA-to-cDNA Kit (Applied Biosystems) and random hexamer or OSM55 (5′-TAGTAGTAGACTCC-3′). For initial identification, we used oligonucleotide primers for SEOV L segment as described (*20*). To obtain the 3′ and 5′ termini genome sequences of SEOV, we performed rapid amplification of cDNA ends (RACE) PCR by using a 3′-Full RACE Core Set and a 5′-Full RACE Core Set (Takara Bio Inc., Kusatsu, Shiga, Japan), according to the manufacturer’s specifications. We purified PCR products by using the LaboPass PCR Purification Kit (Cosmo Genetech, Seoul, South Korea). We performed sequencing in both directions of each PCR product by using the BigDye Terminator v3.1 Cycle Sequencing Kit (Applied Biosystems) on an automated sequencer (Applied Biosystems).

### Multiplex PCR–Based NGS

We designed multiplex PCR primers for SEOV L, M, and S segments and amplified cDNA by using primers (Technical Appendix 1) and primer mixtures and Solg 2× Uh-Taq PCR Smart Mix (Solgent, Daejeon, South Korea), according to the manufacturer’s instructions. We performed the first and second enrichments in a 25-μL reaction mixture containing 12.5 μL of 2× Uh pre-mix, 1 μL of cDNA template, 10 μL of primer mixture, and 1.5 μL of distilled water. Initial denaturation was at 95°C for 15 min, followed by 40 cycles or 25 cycles at 95°C for 20 s, 50°C for 40 s, and 72°C for 1 min, and a final elongation at 72°C for 3 min.

We prepared multiplex PCR products by using the TruSeq Nano DNA LT Sample Preparation Kit (Illumina, San Diego, CA, USA) according to the manufacturer’s instructions. We mechanically sheared samples by using an M220 focused ultrasonicator (Covaris, Woburn, MA, USA). The cDNA amplicon was size-selected, A-tailed, ligated with indexes and adaptors, and enriched. We sequenced libraries by using the MiSeq benchtop sequencer (Illumina) with 2 × 150 bp and a MiSeq reagent V2 (Illumina). We imported and analyzed Illumina FASTQ files by using EDGE (*21*).

### Phylogenetic Analysis

We aligned and edited virus genome sequences by using the multiple sequence alignment with high accuracy and high throughput algorithm (*22*). We generated phylogenetic trees by using the maximum-likelihood method in MEGA version 6.0 (*23*) and models for analysis according to the best fit substitution model (TN93 + gamma + invariate for L segments, general time reversible + gamma + invariant for M segments, and T92 + gamma for S segments). We assessed support for topologies by bootstrapping for 1,000 iterations. The prototype strain used, SEOV 80-39, was isolated from *R. norvegicus* rats captured in Seoul in 1980.

## Results

### Retrospective Analysis of HFRS Patient Specimens

We found that specimens collected in 2002 from 6 HFRS patients were positive for SEOV by ELISA (J.-W. Song and H. Kariwa, unpub. data). We confirmed that the HFRS specimens were serologically positive for SEOV by IFA (Table 1). Titers of SEOV-specific antibody ranged from 1:128 to 1:4,096. Reverse transcription PCR detected the partial sequence of L segment (nt 2946–3335) from 2 HFRS patients (Hu02-180 and Hu02-258).

**Table 1:** Laboratory diagnosis of samples from patients with SEOV-induced HFRS, South Korea, 2002*

Sample	Onset date	Collection date	IFA titer†	Reverse transcription quantitative PCR result‡
SEOV Hu02-112	Unknown	Unknown	1:128	–
SEOV Hu02-180	2002 Feb 9	2002 Feb 15	1:1,024	+
SEOV Hu02-258	2002 Feb 28	2002 Mar 7	1:4,096	+
SEOV Hu02-294	Unknown	Unknown	1:4,096	–
SEOV Hu02-529	2002 May 8	2002 May 13	1:2,048	–
SEOV Hu02-668	2002 May 30	2002 Jun 18	1:128	–

*HFRS, hemorrhagic fever with renal syndrome; Hu, human; IFA, indirect immunofluorescence antibody test; SEOV, Seoul virus; +, positive; –, negative. †For IgG against SEOV. Antibody titration was tested by using serial 2-fold dilutions starting at a serum dilution of 1:32. ‡For large segment RNA using hantavirus genus–reactive primers.

### Epidemiologic Surveillance of *R. norvegicus* Rats

We collected 1,269 *R. norvegicus* rats in urban HFRS-endemic areas in South Korea, including the city of Seoul (1,226/1,269) and Gyeonggi (40/1,269), Gangwon (1/1,269), and Jeollanam (2/1,269) Provinces (Table 2). A total of 76 (6.2%) of 1,226 rats collected in Seoul were serologically positive for SEOV. However, we found IgG against SEOV in only 1 (2.3%) of 43 rats collected from the other areas, including Gyeonggi, Gangwon, and Jeollanam Provinces. We detected SEOV RNA in 13 (16.9%) of 77 seropositive *R. norvegicus* rats. Serologic prevalence of SEOV in male rats (7.5%, 43/576) was not significantly different from that in female rats (5.0%, 34/684) (p = 0.0763 by χ^2^ test). Serologic prevalence of SEOV in rats by weight (age) was 6.1% (21/342) in those weighing <50 g, 5.9% (22/374) in those weighing 51–100 g, 5.8% (30/521) in those weighing 101–200 g, and 13.8% (4/29) in those weighing 201–300 g. Seasonal prevalence of SEOV infection in rats was 5.8% (11 of 190) in spring (March–May), 4.4% (19/433) in summer (June–August), 6.4% (27/420) in fall (September–November), and 10.0% (19/190) in winter (December–February).

**Table 2:** Serologic and molecular prevalence of SEOV in *Rattus norvegicus *rats, by metropolitan area/province, sex, weight, and season, South Korea, 2000–2016*

Characteristic	No. rats	Prevalence of IgG against SEOV, no. positive/no. tested (%)	SEOV RNA, no. positive/no. tested (%)
Region, n = 1,269			
Seoul	1,226	76/1,226 (6.2)	13/76 (17.1%)
Gyeonggi Province	40	1/40 (2.5)	0/1
Gangwon Province	1	0/1 (0)	ND
Jeollanam Province	2	0/2 (0)	ND
Sex, n = 1,269			
M	576	43/576 (7.5)	9/43 (21.0)
F	684	34/684 (5.0)	4/34 (11.8)
Unknown	9	0/9 (0)	0/9 (0)
Weight, g, n = 1,269			
<50	342	21/342 (6.1)	2/21 (9.5)
51–100	374	22/374 (5.9)	3/22 (13.6)
101–200	521	30/521 (5.8)	7/30 (23.3)
201–300	29	4/29 (13.8)	1/4 (25.0)
Unknown	3	0/3 (0)	ND
Season, n = 1,269			
Spring, Mar–May	190	11/190 (5.8)	0/11 (0)
Summer, Jun–Aug	433	19/433 (4.4)	2/19 (10.5)
Fall, Sep–Nov	420	27/420 (6.4)	6/27 (22.2)
Winter, Dec–Feb	190	19/190 (10.0)	5/19 (26.3)
Unknown	36	1/36 (2.8)	0/1 (0)

*ND, not determined; SEOV, Seoul virus.

### SEOV RNA Loads in Tissues from Seropositive *R. norvegicus *Rats

**Figure 1:**
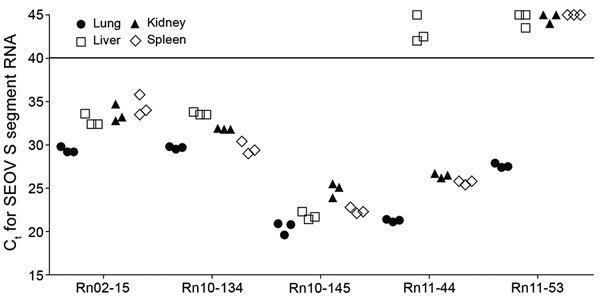
Measurement of SEOV RNA loads in different tissues of *Rattus norvegicus* rats, South Korea, 2000–2016. C_t_ values were determined for SEOV small segment RNA in lung, liver, kidney, and spleen tissues obtained from 5 rats positive for SEOV IgG and SEOV RNA. Solid horizontal line indicates assay cutoff value. C_t_, cycle threshold; S, small; SEOV, Seoul virus.

To measure viral load of SEOV RNA in *R. norvegicus* rats, we performed real-time qPCR for seropositive samples from lungs, livers, kidneys, and spleens (Figure 1). Viral load for SEOV showed ranges from tissues of 5 rats (Rn02-15, Rn10-134, Rn10-145, Rn11-44, and Rn11-53) that were positive by serologic and molecular screening (IFA+ PCR+). Rat Rn10-145 showed the highest amount of SEOV RNA in all tissues, followed by rats Rn02-15 and Rn10-134. Rat Rn11-44 showed the highest amount of SEOV RNA in all tissues except liver. Rat Rn11-53 showed the highest amount of SEOV RNA load in lung tissues, but virus RNA was not detectable in liver, kidney, and spleen tissues.

### Multiplex PCR–Based NGS for Retrospective HFRS Patient and *R. norvegicus* Rat Specimens

We determined viral loads for HFRS patient specimens by using real-time qPCR. Cycle threshold (C_t_) values ranged from 27.5 to 36.8 (Table 3). To perform multiplex PCR–based NGS for SEOV, we designed multiplex PCR primers to amplify every 150-bp sequence for the entire SEOV tripartite genome. We recovered genomic sequences of SEOV from 6 SEOV-positive patient samples. We sequenced human sample Hu02-258, which showed the highest viral load (lowest C_t_ value), for 99.6% of the L segment, 99.7% of the M segment, and 91.6% of the S segment. Recovery rates for SEOV genomic sequences from samples Hu02-180 and Hu02-529 showed a correlation with viral loads. Samples Hu02-112, Hu02-294, and Hu02-668 showed high recovery rates of SEOV S and M segments despite lower viral loads (highest C_t_ values). However, the L segment showed relatively low coverages (85.0% for Hu02-180, 68.2% for Hu02-294, and 72.7% for Hu02-668).

**Table 3:** Quantitation and multiplex PCR–based NGS coverages of SEOV RNA, South Korea, 2000–2016*

Species and sample			SEOV genomes, % coverage‡		
	Origin	C_t_†	L segment, nt 1–6530	M segment, nt 1–3651	S segment, nt 1–1769
Human					
SEOV Hu02-112	Serum	36.0	85.0	93.2	86.0
SEOV Hu02-180	Serum	28.0	86.2	93.2	87.9
SEOV Hu02-258	Serum	27.5	99.6	99.7	91.6
SEOV Hu02-294	Serum	36.5	68.2	94.5	97.7
SEOV Hu02-529	Serum	32.1	94.9	93.8	97.7
SEOV Hu02-668	Serum	36.8	72.7	93.4	85.1
*Rattus norvegicus *rats					
SEOV Rn10-134§	Lung	27.3	99.6	99.2	98.9
SEOV Rn10-145§	Lung	16.1	99.7	99.7	99.4
SEOV Rn11-44§	Lung	21.3	99.1	99.5	98.3
SEOV Rn11-53§	Lung	27.6	99.6	99.7	98.8

*C_t_, cycle threshold; Hu, human; L, large; M, medium; NGS, next-generation sequencing; Rn, rat; S, small; SEOV, Seoul virus. †Determined by real-time quantitative PCR specific for SEOV S segment. ‡Genome coverages were calculated by obtained consensus sequence matching to genome positions of SEOV 80-39 strain (GenBank accession nos. NC_005238, NC_005237, NC_005236). §Whole-genome sequences of SEOV L, M, and S segments were obtained by 3′ and 5′ rapid amplification of cDNA ends PCR.

Using total RNA extracted from rat lung tissues, we determined viral loads by using real-time qPCR. C_t_ values ranged from 16.1 to 27.6. We applied multiplex PCR–based NGS for whole-genome sequencing of 4 SEOV strains in the IFA+ PCR+ rats captured in South Korea during 2000–2016. Coverage of genomic sequences of SEOV was 99.1%–99.7% for L segments, 99.2%–99.7% for M segments, and 98.3%–99.4% for S segments. We observed a correlation between C_t_ values and multiplex PCR–based NGS coverages (Technical Appendix 2). Whole-genome sequences from Rn10-134, Rn10-145, Rn11-44, and Rn11-53 were obtained with termini sequences of 3′ and 5′ ends. SEOV sequences were deposited in GenBank (accession nos. MF149938–MF149957).

### Global Diversity of SEOV

We generated phylogenetic trees by using nearly complete genome sequences of SEOV and the maximum-likelihood method. Phylogenetic analysis demonstrated distinct phylogenetic groups (groups A–F). Group A contained SEOV strains from northeastern and southeastern China and an SEOV strain from North Korea. Group B contained SEOV strains from Southeast Asia (Singapore and Vietnam) and France. Group C contained SEOV strains from South Korea and Japan and SEOV strains Tchoupitoulas from Louisiana in the United States. Group D contained an EOV strain from Jiangxi and Hubei Provinces in southeastern China. Group E contained strains from the United Kingdom and the United States (New York, NY, and Baltimore, MD). Group F contained SEOV strains from mountainous areas in southeastern China.

**Figure 2:**
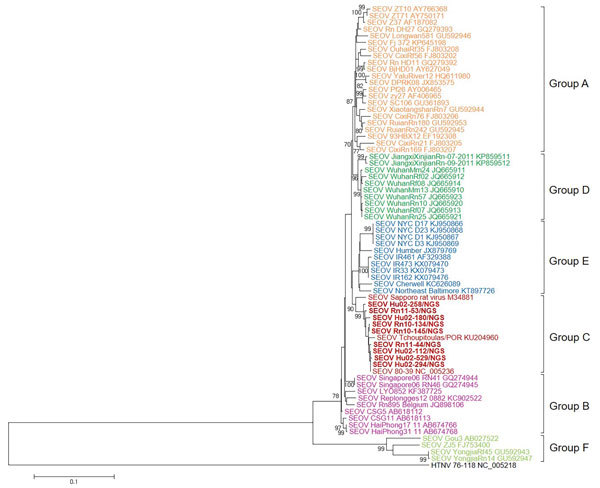
Phylogenetic analysis of SEOV small RNA segments, South Korea, 2000–2016, and reference strains. A phylogenetic tree was generated by using the maximum-likelihood method with the T92 + gamma distribution model of evolution and alignment of small RNA segment sequences (nt 193–1332) of SEOV strains. Colored groups indicate the areas where SEOV strains were identified: group A, northeastern and southeastern China and North Korea; group B, Europe (France and Belgium) and Southeast Asia (Vietnam and Singapore); group C, South Korea, Japan, and the United States; group D, southeastern China; group E, United Kingdom and the United States; group F, mountainous areas in southeastern China. Bold red indicates SEOV strains sequenced in this study. Topologies were evaluated by bootstrap analyses of 1,000 iterations. Numbers along branches are bootstrap values. GenBank accession numbers are provided. Scale bar indicates nucleotide substitutions per site. SEOV, Seoul virus.

We obtained 9 genome sequences of SEOV S segments from HFRS patients and *R. norvegicus* rats. Phylogenetic analysis of SEOV S segments showed that group A formed a monophyletic lineage with group D (Figure 2). Group C genetically clustered with group E. The phylogeny of group B was distinct from those of groups A, C, D, and E. Group F from mountainous areas in China formed a lineage that was independent from the other groups obtained from rats collected in urban areas.

**Figure 3:**
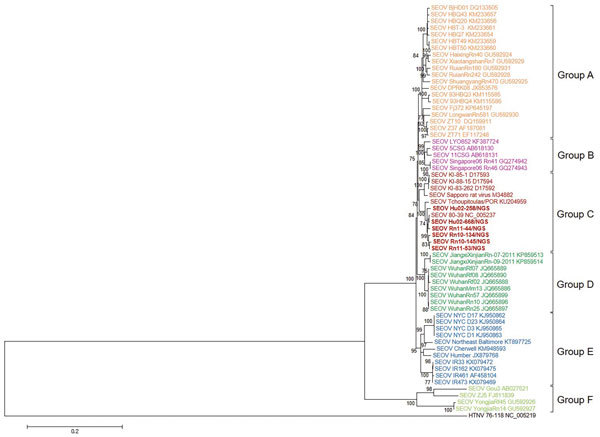
Phylogenetic analysis of SEOV medium RNA segments, South Korea, 2000–2016, and reference strains. A phylogenetic tree was generated by using the maximum-likelihood method with the general time reversible + gamma + invariant model of evolution and alignment of medium segment sequences (nt 47–3430) of SEOV strains. Colored groups indicate the areas where SEOV strains were identified: group A, northeastern and southeastern China and North Korea; group B, Europe (France and Belgium) and Southeast Asia (Vietnam and Singapore); group C, South Korea, Japan, and the United States; group D, southeastern China; group E, United Kingdom and the United States; group F, mountainous areas in southeastern China. Bold red indicates SEOV strains sequenced in this study. Topologies were evaluated by bootstrap analyses of 1,000 iterations. Numbers along branches are bootstrap values. GenBank accession numbers are provided. Scale bar indicates nucleotide substitutions per site. SEOV, Seoul virus.

We obtained 6 genome sequences of SEOV M segments from HFRS patients and *R. norvegicus* rats (Figure 3). Phylogenetic analysis of SEOV M segments showed distinct phylogenetic clusters (groups A–F). These phylogenetic patterns showed that M segments of SEOV had genetic heterogeneity when compared with S segments.

**Figure 4:**
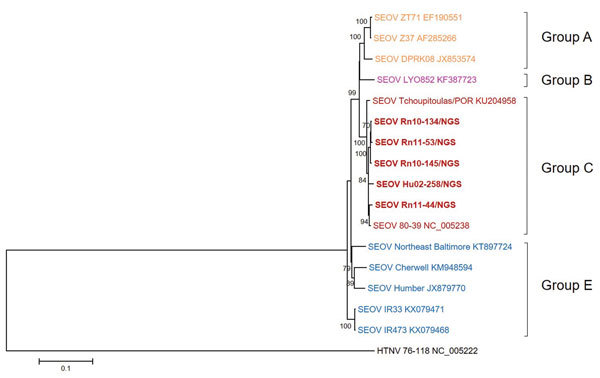
Phylogenetic analysis of SEOV large RNA segments, South Korea, 2000–2016, and reference strains. A phylogenetic tree was generated by using the maximum-likelihood method with the TN93 + gamma + invariant model of evolution and alignment of large segment sequences (nt 1–6510) of SEOV strains. Colored groups indicate the areas where SEOV strains were identified: group A, southeastern China and North Korea; group B, Europe (France); group C, South Korea and the United States; group E, United Kingdom and the United States. Bold red indicates SEOV strains sequenced in this study. Topologies were evaluated by bootstrap analyses of 1,000 iterations. Numbers along branches are bootstrap values. GenBank accession number are provided. Scale bar indicates nucleotide substitutions per site. SEOV, Seoul virus.

We obtained and phylogenetically analyzed 5 SEOV L segments (Figure 4). The SEOV L segment from an HFRS patient and *R. norvegicus* rats captured in South Korea clustered to form a monophyletic group with SEOV 80-39. SEOV strains from China belonged to a genetic lineage with SEOV DPRK08 from North Korea. SEOV strains from the United Kingdom and Baltimore formed a close phylogenetic group. SEOV IR33 and IR473 obtained from laboratory outbreaks in the United Kingdom were independent from other SEOV strains.

## Discussion

NGS is a robust tool for obtaining extensive genetic information and completing whole-genome sequences (*18*). However, molecular enrichment plays a critical role in amplification of pathogen genome sequences from clinical or animal specimens. Our previous study showed recovery of nearly whole-genomic sequences of HTNV from HFRS military patients by using virus-targeted molecular enrichment (*19*).

In this study, whole-genomic sequencing of SEOV, an etiologic agent of mild HFRS worldwide, was applied to samples from retrospective HFRS patients and seropositive *R. norvegicus* rats by using multiplex PCR–based NGS. Nearly whole-genome sequences of SEOV tripartite RNA, on the basis of SEOV 80-39 (prototype strain), corresponded to viral loads of patient serum samples and rat lung tissues. Phylogenetic analyses of the genome sequence of SEOV tripartite RNA supported worldwide distributions of SEOV and identified 6 genetic lineage groups. Group A contained SEOV strains from northeastern and southeastern China and North Korea. Group B contained SEOV strains from Singapore and Vietnam in Southeast Asia and Lyon in France. Group C contained SEOV strains originating primarily in South Korea and Japan and an SEOV strain from Louisiana in the United States. Group D consisted of SEOV strains from southeastern China, including Jiangxi and Hubei Provinces. Group E contained SEOV strains from the United Kingdom and eastern United States (New York and Baltimore) and formed a monophyletic lineage. Group F contained SEOV strains from mountainous areas in southeastern China (*24*).

SEOV originated in China and spread worldwide during movement of rats coincidently with human activities (e.g., commercial trade, travel, and migration by railways and through seaports) (*24*). The close genetic relationship of SEOV in South Korea and Japan was probably caused by geographic distance and historical activities (e.g., commerce and occupation by Japanese forces). The genetic lineage containing strains from Southeast Asia and France might have originated during colonization or on trade routes that extended distribution of SEOV-infected rats (*25*). Recently, SEOV outbreaks have been reported in the United Kingdom and United States. Clinical cases showed that SEOV infections were identified among pet owners, breeders, and distributors (*26*). The genetic relationship of SEOV between counties probably reflects movement of rats associated with the animal pet market.

The prevalence of hantaviruses (e.g., HTNV and Imjin virus [MJNV]) in natural reservoir hosts has showed sex- and weight (age)–specific differences (*27*,*28*). However, in our study, the incidence of SEOV in *R. norvegicus* rats was not dependent on sex and weight (age). Epidemiologic differences in hantavirus infections between *A. agrarius* and *R. norvegicus* rats might be, in part, caused by ecologic differences, reservoir host distributions, and behavior (e.g., association with humans) (*29*). Seasonal circulation of SEOV infection was maintained over 1 year, suggesting an enzootic infectious cycle. These observations might suggest that preventive strategies for disease risk mitigation focus on limits of rat populations all year.

Our previous study demonstrated differential amounts of HTNV RNA in lung, kidney, liver, and spleen tissues of rodents collected in areas in which HFRS is prevalent (*30*). In addition, the genomic RNA load of MJNV, a shrewborne hantavirus, showed various patterns in different tissues in nature (*28*). IFA+ PCR+ shrews showed high and various loads of MJNV RNA in all tissues. MJNV RNA from IFA– PCR+ shrews was detected in lung but not in kidney, liver, or spleen tissues, indicating an early phase of infection before MJNV-specific IgG was produced (*31*,*32*). In our study, rats Rn02-15, Rn10-134, and Rn10-145 showed various amounts of SEOV RNA in all tissues. Rat Rn11-44 had high levels of SEOV RNA in all tissues except the liver. Virus RNA in rat Rn11-53 might reflect the early phase of SEOV infections because of highest viral load in lung tissues but not other tissues. Patterns of SEOV RNA loads might indicate systemic infections in nature and active circulation of virus among rat populations in urban HFRS-endemic areas.

Diversity of virus genomes results from genomic variation or exchanges (*33*). RNA viruses show high mutation rates caused by deficiencies in proofreading by virus polymerases. Genomic variation also results from a mechanism of host immune evasion (*34*,*35*). Genetic exchanges, such as reassortment and recombination, lead to the generation of divergent virus progeny (*36*). Our previous studies identified reassortment and recombination of hantaviruses, including HTNV and MJNV, in nature (*19*,*28*,*37*). Using nearly complete sequences of SEOV S, M, and L segments, phylogenetic analyses demonstrated that S segments of group A SEOVs formed a cluster with those of group D SEOVs and that L and M segments of group A SEOVs showed a close phylogenetic relationship with those of group B SEOVs. The S segment of group C SEOVs grouped phylogenetically with group E SEOVs. However, L and M segments of group C SEOVs formed a distant genetic cluster from those of group E SEOVs. Phylogenetic analysis of SEOV S segments showed a differential pattern from that of SEOV M segments, indicating a genome organization compatible with genetic exchanges in nature. To clarify genetic events among SEOV worldwide, whole-genome sequences of the SEOV L segment need to be investigated. Application of multiplex PCR–based NGS will be useful in elucidating phylogenetic patterns of the SEOV L segment.

In conclusion, this epidemiologic survey of *R. norvegicus* rats in urban HFRS-endemic areas of South Korea identified the prevalence and distribution of SEOV. We applied multiplex PCR–based NGS to whole-genome sequencing of SEOV tripartite RNA from retrospective serum samples from HFRS patients and rat tissues. Phylogenetic analyses demonstrated the global distribution and genetic diversity of SEOV on the basis of nearly complete genome sequences. This study provides useful information for SEOV-based surveillance, disease risk assessment, and mitigation against hantavirus outbreaks.

Technical Appendix 1. Seoul virus primer sequences used for multiplex PCR−based next-generation sequencing.

Technical Appendix 2. Additional information on multiplex PCR−based next-generation sequencing and global diversity of Seoul virus in humans and rats.
