# Supplementary material for: Multiplex PCR−Based Next-Generation Sequencing and Global Diversity of Seoul Virus in Humans and Rats
Source: Emerg Infect Dis. 2018 Feb;24(2):249–57. doi: 10.3201/eid2402.171216 (PMC5782898; doi:10.3201/eid2402.171216)
Supplement: Supplementary file 2 — Technical Appendix 2. Additional information on multiplex PCR−based next-generation sequencing and global diversity of Seoul virus in humans and rats. [file 17-1216-Techapp-s2.pdf]

Article DOI: https://doi.org/10.3201/eid2401.171216

Multiplex PCR–Based Next-Generation Sequencing and Global Diversity of Seoul Virus in Humans and Rats

Technical Appendix 2

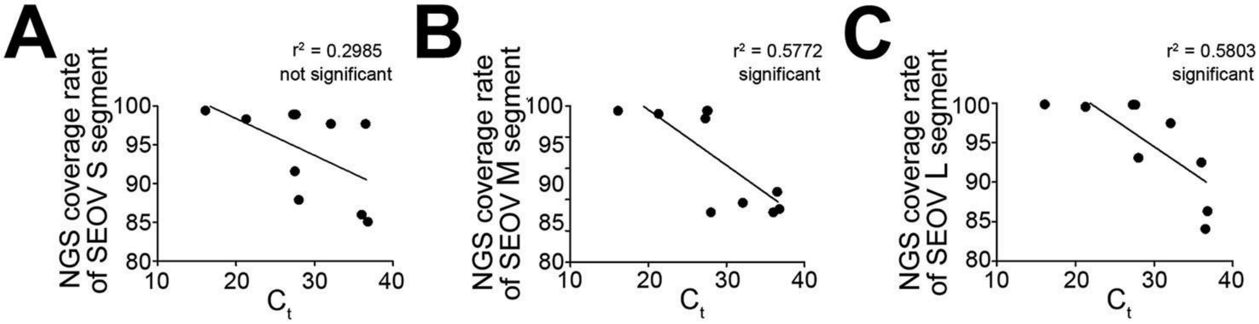

Technical Appendix Figure. Correlation of genomic sequencing of Seoul virus (SEOV) between cycle threshold (C_t) values and multiplex PCR-based next-generation sequencing (NGS) coverages. A) S segment; B) M segment: C) L segment. Correlation of the genomic sequencing of SEOV between C_t values of real-time PCR and multiplex PCR-based NGS coverages was analyzed by using GraphPad Prism version 5.0 (GraphPad Software, San Diego, CA, USA). L, large; M, medium; S, small.
